# Learn Today–Apply Tomorrow: The SMART Pharmacist Program

**DOI:** 10.3390/pharmacy8030139

**Published:** 2020-08-06

**Authors:** Michael J Rouse, Arijana Meštrović

**Affiliations:** 1 International Services Program, Accreditation Council for Pharmacy Education (ACPE), Chicago, IL 60603, USA; 2 Pharma Expert Consultancy and Education, Deščevec 56, 10040 Zagreb, Croatia; arijana.mestrovic@pharmaexpert.hr

**Keywords:** continuing professional development, SMART Pharmacist, pharmacist portfolio, competencies, indicators of quality, pharmacy services, pharmacy education

## Abstract

The SMART Pharmacist Program was initiated by the Accreditation Council for Pharmacy Education (ACPE) and Pharma Expert in 2014. It was designed to introduce a new continuing education model for pharmacists for the Turkish Pharmacists’ Association, and to support development of competencies for future practice. After successful implementation in Turkey, the Program spread to 16 additional countries. To assure quality, globally adopted and validated tools and best practices were used, respecting the national context. National competency frameworks and quality indicators for pharmaceutical care delivery were developed. Pharmacists’ learning portfolios were introduced and patient care modules created. Under the sub-title “Learn Today—Apply Tomorrow,” the changes in practice were introduced under the leadership of national host organizations. The Program showed an impact on the patient level in several countries, especially in areas of patient care in Asthma and Chronic Obstructive Pulmonary Disease (COPD), Hypertension and Dyslipidemia, Diabetes, and the patient care process in general (e.g., identifying drug-related problems, improving patient safety, collaborating with medical doctors). Changes are visible at the individual (pharmacists) and organizational levels. Barriers and facilitators to the change-management process during Program implementation are identified. In some countries, the Program is recognized as one of the most important initiatives in pharmacy education and practice, with visible support of national medicines agencies, academia, government, and WHO regional offices.

## 1. Introduction

There have been many advances in the Pharmacy profession over the past several decades, mainly in the areas of education and practice. Innovative concepts such as clinical pharmacy, pharmaceutical care, self-directed lifelong learning, and patient-centered care have been introduced in the profession. Good concepts, such as these, are essential to advancing a profession, but not sufficient. New ideas have been embraced and implemented by *Innovators* and *Early Adopters* (Rogers’ classification) [1,2] resulting in “pockets of excellence,” but deep pervasive change, which requires strategic profession-wide collaboration and leadership, and buy in and implementation by the *Early Majority* and *Late Majority*, has arguably not yet been achieved. Many factors contribute to this situation; some within the profession and some external to the profession. The Introduction provides important context, describing developments—both successes and challenges—to explain how we have reached this point in the profession.

### 1.1. Advances in Pharmacy Practice

The concept of “clinical pharmacy”—with expanded roles for pharmacists in the care of patients—emerged in the 1960s and was implemented in several countries, primarily in institutional settings [3]. However, these changes initially reflected “pockets of excellence” rather than deep, pervasive change in the practice of pharmacy [4], and product-related services, focused mainly on manufacturing, distribution, and dispensing were the most common role for pharmacists in community, institutional, and other settings. In the 1980s and early 1990s, Doug Hepler and Linda Strand advocated changes in pharmacy education and practice, and articulated the “pharmaceutical care” model [5,6,7]. Pharmaceutical care centered on pharmacists identifying, preventing, and resolving drug-related problems, thereby taking more responsibility and accountability for the outcomes of care; the goals of such care being agreed collaboratively between the patient, the prescriber, the pharmacist, and any other key players.

Pharmaceutical care was embraced by many as a global vision for the practice of pharmacy, but while few within the profession of pharmacy disagreed with it as a concept, implementation of the model posed several challenges. Business, payment, practice models and workflow were still product-focused. Pharmacists were not widely recognized as providers of patient care services, hence there were few, if any, mechanisms by which pharmacists or their institutions could be paid to provide the services needed to achieve the desired goals of medication therapy. Many within the profession were frustrated by the slow pace of change. In presenting the Harvey A.K. Whitney Award Lecture in 2010, Hepler referred to “A Dream Deferred” and stated “We pharmacists have many dreams. Our biggest dream is to become a fully clinical profession” [8]. In his address, Hepler expressed frustration that “the dream of pharmacy as a clinical profession has been deferred for too long”. This despite the groundwork that had been done, and the fact that provision of pharmaceutical care: is feasible in many settings; has been accepted by patients, physicians, and nurses; and has demonstrated significantly improved outcomes of drug therapy at an equal or lower total cost of care. Hepler went on to say “Achieving our dream of universal clinical practice will depend on the choices that pharmacists make. Every strategic, policy and clinical decision must pass the test of whether it will improve the safety and effectiveness of drug therapy and, ultimately, our patients’ quality of life.”

At the same time, many other changes in society, medicine, and technology were impacting the practice of pharmacy; these included: changing patterns of disease and morbidity; ageing populations with a high reliance on medications to manage chronic diseases; more complex and costly medications and biologic agents; trends in self-medication, new business models (e.g., emergence and growth of pharmacy chains and non-traditional locations); inter-professional practice and team-based care; spiraling health care costs; and growing concerns about the adequacy of education of health professionals, practice models, and patient safety, including harm caused by errors and sub-optimal use of medications [9,10,11]. In 2011, Dorman stated that despite the spectacular gains in the breadth and depth of biomedical knowledge, the potential of these gains has been limited by inadequate, inequitable, and inefficient translation of knowledge and skills to the health care workplace. He called for radical transformation of the continuing medical education system [12].

In its 2000 White Paper, the American College of Clinical Pharmacy (ACCP) discussed pharmacy’s changing philosophy of practice and factors influencing the evolution of professional roles and responsibilities and made recommendations regarding preparation needed for future roles and qualifications for practice [13]. ACCP made several conclusions (authors’ summarization):

- Profession-wide retraining will be needed for pharmacists to assume true patient care roles;
- Structured and systematic postgraduate education experiences (i.e., “certificate programs”) should provide much of the retraining that will be needed by the current pharmacist workforce;
- Inadequate mechanisms are currently available to accomplish the retraining necessary for these practitioners to fulfill new clinical practice roles;
- Collectively, the clinical pharmacy practice and pharmacy education communities possess the expertise necessary to create new, practical, and valid means of retraining pharmacists for emerging patient care roles.; however, these sectors of the profession have not yet fully committed to partnering with community pharmacy to create effective, appropriately rigorous retraining mechanisms;
- A broad-based, inclusive planning process involving all pharmacy organizations and associations will be necessary to address the profession’s vast retraining needs.
- Pharmacy faculty and clinical practitioners must make the commitment to provide the expertise and cooperation necessary to develop efficacious education and training programs that can enhance the clinical practice abilities of community pharmacists.

### 1.2. Advances in Pre-Licensure Pharmacy Education

In the United States, many of the early pioneers of the clinical pharmacy movement came from the pharmacy education sector, at institutions such as the University of Michigan and the University of Kentucky [14]. Around the same time, several pharmacy schools started to expand the clinical focus in their curriculums and increase the number of clinical faculty members. The University of Southern California (1950) and the University of California San Francisco (1955) introduced innovative Doctor of Pharmacy (PharmD) degree programs, with several other schools following in the late 1960s [15]. Pharmacy education in the United States and several other countries started to undergo a major transformation to prepare pharmacy graduates for their new roles. In 1992, the American Association of Colleges of Pharmacy (AACP) voted to approve the PharmD as the only professional degree in pharmacy [16] and in 1997, the U.S. national accreditation body, the Accreditation Council for Pharmacy Education (ACPE), announced that with effect from 2000 it would no longer accredit Bachelor of Science programs, and all colleges of pharmacy had to convert to the PharmD. In parallel with changes in pharmacy education, structured post-graduate training programs for pharmacists started to emerge, such as residency programs in the USA, for which accreditation standards were introduced by the American Society of Health-System Pharmacists (ASHP) in 1963. Initially, residencies focused on leadership, hospital administration, and traditional pharmacy operations, but now are mainly focused on areas of clinical practice. Residencies are primarily hospital-based, but in the 1990s, community pharmacy-based residencies started to expand. Similar trends were emerging in Australia, Canada, and Europe. 

### 1.3. Changes in Continuing Pharmacy Education and Regulation

With transformation of pre-service education and expanding roles and responsibilities for practicing pharmacists—albeit primarily in institutional settings—there was a growing awareness of the need for maintaining the continuing competence of pharmacists to practice. In the 1960s and 1970s, several state boards of pharmacy in the USA (pharmacy regulators) started to introduce mandatory continuing education (CE) for maintenance of licensure. In the mid-1970s, the National Association of Boards of Pharmacy (NABP) adopted a resolution on mandatory CE for re-licensure, and the American Pharmaceutical Association-American Association of Colleges of Pharmacy (APhA-AACP) Task Force on Continuing Competence in Pharmacy (1972–1974) concluded that CE was the best available mechanism for assuring pharmacists’ ongoing proficiency [17]. In 1974, the APhA Board of Trustees recommended that ACPE be requested to develop a system of accreditation for CE, and the following year ACPE introduced accreditation standards for CE providers.

Following the conclusions of the 1972–1974 Task Force, it was agreed that the purpose of CE was the improvement of patient care and health maintenance and the enrichment of the practitioner’s career [18]. It was stressed that CE structures being implemented at that time should be recognized as “transitional mechanisms to be used until means are developed to evaluate the competence of the individual pharmacist in the performance of his (sic) professional responsibilities.” Additionally: “It is this competence to perform, which will not be the same for each type of pharmaceutical practice, that eventually must be measured and evaluated.” Over the next forty years, several changes were introduced to improve the quality of CE activities [19]. The core regulatory model (participation in CE), however, remained the same, as systems to evaluate the competence of individual pharmacists in the performance of their professional responsibilities—despite calls for their implementation—did not materialize [11].

Competency in pharmacy is the individual ability to make deliberate choices from a repertoire of behaviors for handling situations and tasks in specific contexts of professional pharmacy practice by using and integrating knowledge and personal values, in accordance with professional role and responsibilities [20]. If behaviour and performance need to be assessed, pharmacists should ideally be observed at their regular working place, where implementation of knowledge, skills, attitudes, and values are required and visible [21]. 

Notwithstanding the recognized limitations of the model, completion of a required number of CE hours or credits became—and remains—the most pervasive model (proxy) for assurance of ongoing competence to practice for pharmacists and other health professionals [22,23]. The model for CE has not substantively changed in the past 50 years, whereas the demands on and expectations of the pharmacy practitioner have evolved tremendously [24]. Some countries, however, still have no requirement for pharmacists to participate in any form of CE in order to maintain their license to practice; this includes Turkey, which will be discussed later in the article.

In the 1990s, there was a growing body of evidence of the limitations of the CE model to achieve the objectives for which it was intended. The nature and format of CE activities most frequently offered indicates that they are predominantly focused on enhancing the knowledge of participants, with less attention given to building skills, attitudes, and values. A year-by-year analysis of a total of 176,198 ACPE-accredited CE activities for the years 2014–2017, indicated that 86–88% of activities were Knowledge-Based, 12–13% were Application-Based, and 1% were Practice-Based, as defined in ACPE Accreditation Standards [25]. All components of competence are, however, essential for contemporary pharmacy practice, and are needed to translate advances in medicine and pharmacy into meaningful changes in healthcare delivery [11,26]. UNESCO’s Fifth Pillar of Learning is “Learning to Transform Oneself and Society,” and for the profession and patient care to move forward, the way pharmacists learn must bring about changes (transformation) in themselves and their practice [4,27,28]. Good ideas and concepts are essential but, in themselves, not sufficient to bring about transformational changes in practice.

**Figure 1:**
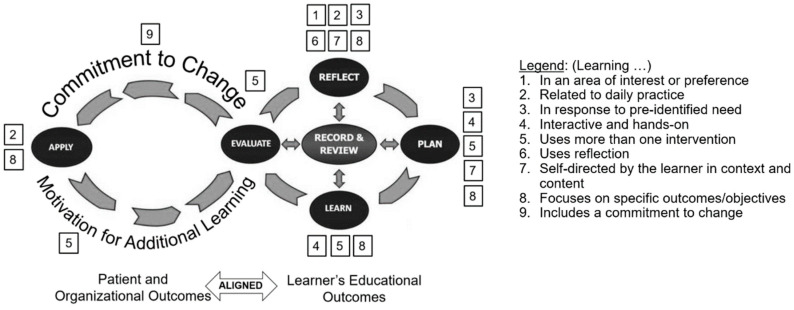
Mapping successful strategies for learning against the Continuing Professional Development (CPD) Cycle. Adapted from original. Copyright © 2020–2014 Accreditation Council for Pharmacy Education. Used with permission.

The regulators of the pharmacy profession in several countries (initially UK, Canada, and New Zealand) started to explore—and later adopted—the Continuing Professional Development (CPD) model [29,30,31]. In the USA at that time, regulators in pharmacy and other health professions retained their traditional CE-based models for maintenance of licensure [26]. Among U.S. pharmacy regulators, a 2011 ACPE survey indicated that awareness about the CPD model was not high and a majority of respondents felt that hours-based CE served as an adequate proxy to assure pharmacist competency [32]. ACPE, however, had recognized the potential for the CPD model to enhance self- directed lifelong learning of pharmacists. Initially, ACPE advocated for the exploration of the CPD model, and later for its adoption to complement the accreditation of CE providers [33,34]. ACPE endorsed the core concepts and elements adopted in other countries, but made several changes to the depiction of the CPD learning cycle. The latest version of the cycle adopted by ACPE (incorporated in Figure 1 below) follows an infinity shape to draw attention to what were considered to be two key differences between the CPD model and traditional CE, namely the need for application of learning and the evaluation of changes made following learning, including the impact thereof [35]. In 2006–7, a CPD pilot was conducted in five U.S. states. The pilot demonstrated the potential benefits of pharmacists adopting a CPD approach [36,37]. The mandatory CE model was, however, well established and accepted by regulators, pharmacists, and CE providers, and there was limited uptake of the CPD model, with only one state (North Carolina) following up at that time to offer pharmacists the option to maintain their license through a CPD mechanism.

ACPE continued to work with stakeholders—including hospitals and health-systems, employers, CE providers, and colleges of pharmacy—to advocate for more widespread support for and uptake of the model [24]. In 2015, ACPE convened a stakeholders’ invitational conference bringing together leaders from within the profession of pharmacy to discuss the future of CE and its accreditation, and CPD was endorsed as a model to enhance lifelong learning for pharmacists [38]. ACPE agreed that its International Services Program (ISP), established in 2011, could play a role internationally in advocating the CPD model and supporting its implementation in other countries. In collaboration with Pharma Expert educational agency, the SMART Pharmacist Program was designed as a practical and sustainable model to address the identified needs of many national organizations that had tried to implement meaningful CPD models in their own countries. It was decided that all elements of the CPD cycle could be embedded in the Program, including some assessment tools and indicators to measure improvements and progress. The methodology of teaching, learning, and enhancing knowledge, skills, and motivation was carefully developed, piloted, and improved during the implementation process, as described in Materials and Methods.

### 1.4. What Gets Measured Gets Done

Table 1 illustrates the challenge in evaluating the outcomes and effectiveness of CE. It is adapted from the work of the Alliance for Continuing Medical Education (ACME; which later became the Alliance for Continuing Education in the Health Professions). ACME described how the outcomes of learning activities can be measured at six levels—the lowest being participation, the highest being population health [39].

In some countries, such as Turkey where the SMART Pharmacy Program (Program) was initiated, there are no regulations requiring pharmacists to participate in CE, and it was the perception of the Turkish Pharmacists’ Association (TPA) at the time that, as a result, there was no meaningful participation in and outcomes from CE activities (“Level 0”). Most countries have a “participation” CE model (Level 1), in which hours, credits, or CE units (CEUs) are counted. The risk of such a model is that pharmacists do little more than participate to comply with the requirement to collect CE points. Most CE providers measure “satisfaction” (Level 2) and this can be helpful for quality assurance and improvement. Only some CE providers assess pre- and post-learning (Level 3) and some CE accreditors require it, but overall direct assessment of learning is probably not widespread. Learners are more frequently encouraged to self-assess their learning. Very few education providers or regulators attempt to measure improvement in performance, patient health, or population health (Levels 4, 5, and 6), yet the latter outcomes are the agreed goals of CE and CPD, and hence should be what is measured [11,18]. Manley et al. emphasized the need to measure the effectiveness of CE/CPD activities, including: the impact on population health; the measurement of perception of satisfaction, competencies, professional performance and healthcare outcomes; and provide information on whether participants were actively engaged, and the desired behavior changes were achieved [40]. Measurement of the enhanced competence of the pharmacist, improvement in quality of services provided (both self-reported), and improved patient outcomes (objective and subjective patient data) are all key elements of the SMART Program.

### 1.5. Successful Learning and Practice Change

Dopp et al. reviewed the findings published in the CE and CME literature regarding effectiveness of CE and summarized them as nine key strategies that have been shown to facilitate meaningful and sustained learning, and be more likely to result in practice changes by the practitioner [37]. Their summary closely aligns with other published findings [40,41,42]. Learning had to be in an area of interest or preference (#1) and related to the daily practice of the learner (#2); learning activities had to be selected in response to a pre-identified need (#3), had to be interactive and hands-on (#4), use more than one intervention, i.e., continuing, not opportunistic (#5), use reflection (#6), be self-directed by the learner in context and content (#7), focus on specific outcomes/objectives (#8), and include a commitment to change (#9). All of these strategies are incorporated in the CPD model. Building on previously published depictions of the CPD cycle [24] based on ACPE’s version, Figure 1 emphasizes that the REFLECT stage (six strategies) and the PLAN stage (five strategies) need specific focus and attention by the learner to achieve optimal results.

### 1.6. Barriers to Change in Practice and Education

There are many factors that influence the rate and nature of change in a profession in the three key sectors of regulation, practice, and education identified in the International Pharmaceutical Federation’s (FIP’s) Global Framework for Quality Assurance of Pharmacy Education [43] It is important, if not essential, for these sectors to communicate and collaborate effectively for the profession to fulfill its mission to society, and to develop and advance [44,45]. There are, unfortunately, many barriers and challenges when it comes to bringing about change; some within the profession, and others external to the profession that are more difficult to influence. The matter is multi-faceted and complex. This paper, in describing the conceptualization, development, and implementation of the SMART Program, focuses on three of the barriers and challenges, one from each of the key sectors; i.e., (a) traditional approaches to continuing **education** of pharmacists that do not optimally motivate and empower pharmacists to change; (b) **practice** and payment models that are still heavily product-focused; and (c) **regulatory** models for maintenance of licensure/registration that more frequently require and measure participation, rather than the maintenance and enhancement of competence, and the improvement of patient care, which are the goals and objectives of CE [18]. In its design and process, the Program strives to bring together all key sectors and stakeholders in the profession to adopt a common vision for change, identify priority needs to be addressed, and collaborate to implement appropriate strategies to achieve the desired outcomes. 

As noted above, medications have become the mainstay of the management of chronic disease. As the global population ages and more patients have access to medication benefits through expanded health insurance benefits or national health systems, the consumption of medication has risen dramatically. This has cost, logistical, and safety implications. Despite a significant increase in the use of generic medications, the increased volume of prescriptions and higher cost of new medications, including specialty drugs and biologics, have led to: a need for better management of resources spent on medications; the emergence of price controls, managed care, formularies, and pharmacy benefit management companies (PBMs); and complicated eligibility and reimbursement models that have to be navigated by pharmacists.

It is encouraging that pharmacists are spending more of their time in the delivery of direct patient care services, but despite the global vision for the delivery of pharmaceutical care, many pharmacists still spend a majority of their time on dispensing-related services and administration [46]. It is likely that the public and many pharmacists still perceive that the primary role of a pharmacist is over when the medication has been dispensed. To the contrary, evidence shows that the value of the pharmacist continues well after the product has been dispensed; e.g., in the better management of the medication use process [47]. The authors pose the question: instead of systems enabling and facilitating medications to make a major contribution to the management of chronic diseases, having product-focused systems—including business and payment models—inadvertently become a barrier to the provision of optimal pharmaceutical care by pharmacists? If pharmacists have to manage very high prescription numbers and their performance metrics are based on volume (not outcomes of care), there are implications for patient safety, as well as pharmacist stress and job satisfaction [46]. What needs to change to enable medications to optimally contribute to patient care? 

### 1.7. The SMART Pharmacist Program Is Conceived

In 2011, ACPE established its International Services Program (ISP) and, as part of its international mission and services, ACPE decided that through ISP, it could assist countries that were interested to introduce or advance models for continuing professional development of pharmacists. This led to ACPE staff conducting several CPD workshops outside the USA. A workshop in Cyprus in April 2014—jointly arranged and presented by Rouse (ACPE) and Meštrović (Pharma Expert)—led to an introduction to the Turkish Pharmacists Association (TPA), which was looking for a meaningful way to engage pharmacists in CE despite the fact that there was no legal requirement for such participation. Discussions with TPA revealed that the issue was broader than just the need for a better CE model. The majority of active pharmacists in Turkey work in community pharmacies, typically as owners of their pharmacy; chains are not allowed. Several threats and challenges were identified: the future sustainability of the business model was uncertain, largely because of government-imposed price controls on medication; pharmacists were anxious about the future and many were demotivated; the practice/business model was a traditional, product-focused retail model; and the scope of practice allowed by regulation was somewhat limited. For the future viability and sustainability of community pharmacists, it was suggested that a new practice model was needed, which would rely more on the expanded provision of professional, patient care services. Pharmacists would need to be motivated and empowered to provide such services, and this would require—most importantly—a different approach to the education and training of practicing pharmacists.

The SMART Pharmacist Program was originally conceived as the SMART Pharmacy Project (“Project”), based on the key principles of *Commitment to Quality* and *Commitment to Change*. “SMART” was chosen not only because of its implied meaning (*intelligent*, *well-presented*), but because it defined what the objectives of the Project should be: **S**pecific (precise about the desired achievement); **M**easurable (quantifiable objectives); **A**chievable (realistic expectations); **R**elevant (aligned with practice and/or organizational goals); and **T**imed (when the objective will be achieved is stated). The CPD model and concepts related to quality of education and competency frameworks for pharmacists, based on FIP tools and resources, were presented to Turkish pharmacists at the national congress in September 2014 [43,48]. Preliminary discussions were held with members of the TPA Board of Directors and staff, to gain a better understanding of the situation (context) in Turkey. In December 2014, a presentation was made to the full TPA Board; it was titled *“SMART Pharmacy—Shaping our future: Innovative model to drive changes in the pharmacy profession.”* The presentation described how SMART Pharmacy was based on FIP’s “Pillars of Quality” model, addressing *Context*, *Structure*, *Process*, *Outcomes,* and *Impact* [43], as follows:

Context:
  - No license renewal required for pharmacists
  - No structured national education plan
  - Competencies not defined on the national level
  - The need for transformation of CE to CPD model of education
  - A commitment to advance pharmacy profession
Structure:
  - Involve stakeholders in pharmacy profession
  - Include leaders and motivators—innovation workshop
  - Train the trainers for pilot project
  - Pilot project
  - Translation of the results (experience and knowledge) on national level
Process:
  - Innovation workshop for leaders
  - Define SMART goals and plan
  - Develop the methodology for change implementation
  - Train-the-Trainer interactive workshop based on CPD principles
  - Pilot project
Outcomes:
  - Development and adoption of the Turkish Competency Framework for Pharmacists
  - A new, competency based educational model for pharmacists
  - A new, visible identity of pharmacy profession
  - Quality assurance of pharmacy CPD educational model
Impact:
  - Stronger role and reputation for TPA
  - Competency development of pharmacists
  - New services for patients
  - Improved self-image of pharmacists
  - Collaborative practice
  - Improved patient safety and outcomes

TPA made a decision to move ahead with the Project, and planning was started. To emphasize how this project would be different from previous attempts to introduce changes in pharmacy practice, the originators of the Project suggested the subtitle: *Learn Today–Apply Tomorrow,* which was accepted and embraced as an important point.

## 2. Materials and Methods 

The concepts, elements, tools and resources needed to deliver the Project were relatively easy to identify. Several had already been developed and adopted by FIP and other international organizations, and implemented to some extent in different countries. It was recognized, however, that the biggest challenge to success was likely to be related to change management, as the Project would entail radical change for pharmacists and other stakeholders. The TPA was the ideal national partner for the Project, as it was able to bring together all key sectors in the profession. In addition, TPA had the vision, respect, credibility, resources, authority, communication skills, influence, and importantly a sense of urgency, essential in change management [49]. Following Kotter’s model, the next steps were to develop the change vision and strategy, communicate for understanding and buy in, and empower others to act.

In January 2015, with its partners, ACPE and Pharma Expert (ACPE/PE), TPA convened an “Innovation Workshop” bringing together the key stakeholders for two days of intensive activities to: introduce key concepts, including the CPD model, FIP’s Pillars and Foundations of Educational Quality, and Needs-Based Education models, indicators of quality of services, competency in pharmacy and its assessment, change and time management, and commitment to change; tools and resources; undertake a SWOT Analysis of Pharmacy in Turkey; identify and agree on priority needs and opportunities that could be impacted by pharmacists; discuss change management principles; and identify the strategies needed to implement the project. It was agreed that the Project would be piloted with a small, selected group of eight of TPA’s Chambers (regional associations under TPA). The method would be to train local trainers, who would in turn train pharmacists in the key concepts and a clinical module identified by the stakeholders that was: a priority healthcare need; feasible to implement; and for which there would be high and easily measurable impact from the pharmacists’ contribution. Asthma and Chronic Obstructive Pulmonary Disease (COPD) were selected for the clinical module.

The Project was not designed as a research study, but as a quality improvement initiative, aimed at improving quality and outcomes of educational activities, enhancing competence and motivation of pharmacists, and improving quality of pharmacy services and patient outcomes. Data collection was not intended to relate to a specific pharmacist or patient, only to provide evidence of what could be achieved when pharmacists adopted the CPD model. Due to different systems and regulations, prior to implementation the host in each country was requested to determine what ethical committee or review board (IRB) approval, and patient and pharmacist consent, if any, was needed. Using the National Institutes of Health (NIH, Bethesda, MD, USA) Office of Extramural Research (OER) decision tool, the Project did not classify as human subjects research because patients’ data were not collected specifically for the Project and no one on the “study team” had access to patient identifiers linked to the data [50].

A TPA Project Team was established to work with ACPE/PE to: identify the eight selected Chambers, develop the communication and marketing strategy, motivate and engage other partners, and coordinate the development or adaptation of the key tools to be used in the Project, namely: (a) the Turkish Pharmacist Competency Framework (adapted from FIP’s Competency Framework); (b) the Quality Indicators for Pharmacy Services (adapted from the European Directorate for the Quality of Medicines and Healthcare, EDQM, Quality Indicators for Pharmaceutical Care) [51]; and (c) a Pharmacist’s CPD Portfolio (based on the ACPE model [52]. In February and March 2015, adaptations to the tools were made and validated by focus groups and expert panels, led by the members of the TPA Project Team.

A three-day Train-the-Trainer Workshop was conducted in April 2015 by Project originators, Rouse and Meštrović, (originators), supported by the TPA Project Team. The first two days focused on concepts, tools, and resources for CPD, as a new and innovative model for CE in Turkey (“CPD Module”); the third day was devoted to patient care in Asthma and COPD. In the clinical module, there was a brief focus and update on theory (disease management and guidelines, medications, therapeutic aspects, case studies, etc.), but most of the training focused on practical issues, such as: demonstration of asthma inhalers (including correct technique), peak flow meters and other devices; practice skills; effective patient interaction, education, and communication; and data collection). Trainers were presented with certificates after completion of the modules.

Over the next weeks, using standardized presentations and materials, the trainers trained a group of volunteer pharmacists from their Chamber (on average 30–40). Participating pharmacists were required to complete the REFLECT portion of the CPD Portfolio, undertake a baseline self- assessment of their competencies (using the new Turkish Competency Framework) and quality of the pharmacy services that they provided (using the National Quality Indicators). As the sub-title of the Project was *Learn Today—Apply Tomorrow*, the next task was to implement the principles, knowledge, and skills in their everyday practice. Over the next five months, pharmacists worked with their Asthma and COPD patients to provide counselling and guidance about their illness, improve inhaler technique, monitor the frequency of inhaler use, provide instruction on and measure peak flow rate (PFR) using a PFR meter, administer the Asthma Control Test (questionnaire), and collect and record the patient data over repeated visits. Some pharmacists also noted any changes in lifestyle or habits, and improvement in quality of life reported by patients. TPA designed the patient data collection forms, which were later developed into an online portal. Trainers were requested to maintain regular contact with participating pharmacists and provide support and encouragement. After approximately six months, follow-up meetings were held, and the pharmacists were requested to repeat the self-assessment of their competencies and quality of services and submit the results, along with the patient data that they had collected.

As presented in Results, although the data sample from the pilot project was relatively small, the outcomes were encouraging. In April 2016, the first results were presented to the Turkish Minister of Health, who was impressed by the data. He encouraged national implementation and suggested that pharmacists should be certified through this process and be paid to provide services such as patient education as “health advisors.” TPA decided to expand the Project in phases to the other Chambers. Based on lessons learned from the pilot, minor changes were made, including the system for data collection, which had proven to be one of the main challenges for pharmacists. Two more presentations of results were organized in 2017 and 2018, as well as a presentation to the National Agency of Medicines in 2017, which became a key supporter of the Project.

A series of train-the-trainer (TTT) workshops were held (April 2015–August 2017) until all 54 TPA Chambers had participated. All TTT workshops were provided by the originators of the Project to ensure quality and consistency of content and methodology. Follow-up workshops were conducted for national coordinators and trainers in April 2017 with approximately 300 trainers participating. A regional coordinators’ meeting was held in June 2018 with more than 120 coordinators present. In 2017, the SMART Pharmacy Project was renamed to SMART Pharmacy Program (“Program”), signifying the success of the Project and the long-term commitment of TPA to continue the Program in Turkey.

The second clinical module, Diabetes, was introduced in 2017, developed in the main by the TPA Education Team, with support and guidance from ACPE/PE, and using the new “SMART Education Model.” To achieve buy in to the Program, several presentations were made at national and regional conferences of pharmacists in Turkey. TPA considered the SMART Program to be the most important and impactful program for the pharmacy profession in Turkey. In 2018, the Program adopted a Turkish name, *Rehber Eczanem*, “My Guiding Pharmacist.” The same year, the TPA Education Team developed new clinical modules for Hypertension and Patient Care. 

### Expansion of the Program to Other Countries

Working with a variety of national partners, the Program was introduced in an additional 16 countries; partner organizations and initiation dates are described in Results. Whenever possible, the same model and format were followed: initial discussions with the partner/host(s); Innovation Workshop involving key stakeholders; development/adaptation and adoption of national frameworks, tools, and resources using focus groups of local experts with guidance from ACPE/PE; TTT workshop(s); standardized training for groups of pharmacists; baseline data collection; several months of implementation of patient services and data collection; follow-up with pharmacists; post- implementation data collection; data compilation and analysis; and then introduction of additional clinical modules. 

All Train-the-trainer sessions were led by the originators of the Program and delivered in English, but in most of the countries they were translated into the national language, using simultaneous translation. The validation process usually included focus groups, and combination of the Delphi and nominal group process to adopt national frameworks and assessment tools. Priority healthcare needs were identified at the Innovation Workshops, usually including up to 60 stakeholders and national opinion leaders. Using the results of SWOT analyses, the participants of the Innovation Workshops focused on the identified opportunities, taking into consideration and voting on the following criteria for inclusion: importance/societal and patient need, potential impact, and feasibility of implementation in the near future; in each criterion using a “high” or “low” rating. The opportunities with the highest overall score were selected.

For several reasons, in the majority of countries, Asthma and COPD was the first clinical module: the diseases are health priorities in most countries; the pharmacotherapy is simple; the services that pharmacists can provide in the Program are well within pharmacists’ regulated scope of practice; and patient outcomes are easy to measure objectively, and can be improved quickly. Other clinical modules developed and implemented include, Diabetes; Hypertension and Dyslipidemia; the Pharmacists’ Patient Care Process (PPCP) combined with Drug-Related Problems (identification, documentation, and interventions). 

In 2019, on the fifth anniversary of the SMART Program, it was renamed as the SMART *Pharmacist* Program, emphasizing the personal, individual dedication and commitment of the pharmacist to learning, applying, and changing pharmacy practice in their own environment.

## 3. Results

### 3.1. Introduction, Educational Activities, and Development of the Program

To date, in its different and expanded formats, the Program has been introduced in 17 countries (Table 2). In four countries, it is hosted by the national pharmacists’ association, in five by academic institutions, in three by the national pharmacists’ chamber (regulators), in two by private organizations or non-governmental organizations (NGOs), in one by a hospital, and in two by the Ministry of Health or national health system.

In eight countries, the Program started with an Innovation Workshop, to understand the national context in which to introduce the new educational model, and to list the top opportunities for introducing new services. In 12 countries, the Program was introduced as a model by conducting a Master Class or SMART Workshop to present the principles of the Program, and explain the methodology and tools used. Both methods of introduction have shown the potential for the Program to develop other activities, as shown in Table 2.

In 11 countries, TTT activities were organized and completed, and in five countries, trainers trained practicing pharmacists. In four countries, this process has just started, or is ready and scheduled to start soon. As a result of the Innovation Workshops and Master Class activities, some of the most commonly identified educational needs were: leadership courses; education of preceptors, teachers, and students about a self-directed life-long learning approach; and clinical modules (pharmacotherapy updates and the patient care process). To address these diverse needs, the Program has expanded to three additional educational streams: the SMART Leadership Academy, the SMART Student Program, and SMART Clinical Pearls. All formats are designed to address all aspects of competence: knowledge, skills, attitudes and values, and they all maintain the key principle: *Learn Today—Apply Tomorrow.*

As a result of the Program, especially based on Innovation Workshop activities including a SWOT analysis, many countries have identified the national *strengths*, *weaknesses*, *opportunities*, and *threats* in the pharmacy profession, as well as the stakeholders that should be included in the change process to advance pharmacy practice and education. Some countries have published reports and conclusions describing those findings and used them as a starting point to initiate important changes.

Further results that have emerged from the Program are: establishment or reinforcement of national documents and tools, such as competency frameworks (Turkey, Montenegro, Serbia, Qatar, and Indonesia) and indicators of quality for services (Turkey, Oman, Indonesia, Qatar, and Montenegro), and their use for pharmacists’ self-assessment during the reflection and evaluation stages of the CPD cycle. Some countries have introduced the SMART Pharmacist Learning Portfolio (Turkey, Montenegro, Serbia, Oman, Qatar, Jordan, Indonesia, and Estonia), and some have developed new guidelines and tools (such as Drug-Related Problems Classification in Oman), or public health campaigns (Turkey, Oman, and Montenegro).

The specific case of Turkey—the first and most successful country to date included in the Program—shows how the national organization can take the lead in adopting and adapting the original elements of the Program to transform it into a country-specific program. TPA expanded the offerings of clinical modules based on the knowledge and experience gained from the Program. Turkey’s results will be described in a separate publication.

### 3.2. Trainers, Participants, and Leaders of the Program

As shown in Table 3, in total, 9460 pharmacists have been trained in various clinical modules, and 6063 in CPD modules based on SMART principles and tools, using standardized learning methodology, materials, and presentations. Of the 6,063 pharmacists trained in CPD, 721 have become trainers themselves, embracing that new role to encourage their colleagues from practice. Notably, only a few of those participants had some prior teaching experience.

All trainers have started to maintain their own learning portfolio and at the TTT workshops have self-assessed their competencies and indicators of quality of services in their own work place. They have identified areas for improvement and started to create their own SMART Learning Objectives.

### 3.3. Data Collection, Interpretation and Presentation

Table 4 shows what was reported from the host organizations following implementation of the Program. In 10 of the 17 countries, data collection on the pharmacist level (scores for self-assessment of competencies and indicators of quality of patient services) was recorded at the beginning of the program, but not in all cases were the data interpreted and included in the summary results. Data collection after each learning cycle was collected only in Turkey, Oman, Montenegro and Qatar, and in all those countries the levels of competencies and quality indicators (QI) showed some improvement. As yet, no results from the Program have been published in a journal, but in several countries, data collection is in progress. Some specific details could play a role in further development of pharmacy services, as the QIs of patient care have shown improvement in categories such as: Patient Safely, Prevention and Public Health, Patient Counselling and Diagnosis, Assessment of Medicines, Interprofessional Collaboration, Follow-Up and Documentation, and Pharmacists’ CPD. Some of those changes were significant in certain countries, as the improvements were most visible in categories that were rated the lowest at the beginning of the Program.

Two examples of improvements of QIs for patient services are from Qatar and Oman. In the SMART Pharmacist’s Learning Portfolio, pharmacists recorded levels of QIs before the learning cycle, and after the learning and application cycles. Scores increased, especially in following categories of nationally-adopted QIs: Follow-Up and Documentation, Interprofessional Collaboration, Patient Safety, and Rational Pharmacotherapy.

### 3.4. Data Collection on the Patient Level

Data from pharmacists reporting on their everyday practice have been collected in four countries to date (Turkey, Montenegro, Oman, and Qatar). In some other countries, data collection is in progress. Results were submitted by host organizations after pharmacists’ interventions in practice. The authors did not collect, compile, nor interpret the raw data to be able to provide more details, but country specific publications are pending with detailed analyses. Summarized results are provided in the manuscript to describe what educational activities could change in practice.

#### 3.4.1. Asthma and COPD

The first clinical module introduced in Turkey was Asthma and COPD. SMART Pharmacists collected data from 873 patients on their first and second visit, measuring the difference in the Asthma Control Test (ACT) scores, Peak Flow Meter (PFM) values, and average frequency of use of salbutamol per week, as possible measures of the impact of pharmacists’ interventions. The average improvement of the Asthma Control Test Score of patients compared with the first visit was 76%, and the average improvement for patients of their Peak Flow Rate (PFR) value compared with the first visit was 44% in the pilot project. In the later stages of the Program, the average PFR values of patients compared to their first visit was increased by 63%. A 30% decrease occurred in salbutamol use by patients who improved their inhalation technique or become more adherent to the therapy. A large number of patients demonstrated incorrect inhalation technique on their first visit.

In Oman, from the sample of 370 patients, on average the decrease in salbutamol use per week was 50%, ACT scores were improved by 28%, and PFR values improved by 18%. In Montenegro, based on the sample of 291 patients, 54% of patients had a better Asthma Control Test score after a second visit.

#### 3.4.2. Patient Care Process

The results in identifying and solving drug-related problems (DRP) with Omani pharmacists’ interventions were observed in the sample of 1018 patients during 2017–18. The interventions suggested by pharmacists for patients with DRPs were accepted by physicians in 85% of cases. In Oman, interprofessional collaboration between physicians and pharmacists grew substantially, and DRPs were completely solved in 70% of recorded cases. More data about frequency of different DRPs were collected, and will be published in a country specific case report.

#### 3.4.3. Diabetes

From the sample of 323 diabetic patients in the SMART Pilot Project in Qatar, 149 had a second documented visit with the pharmacist. Out of those, 48% of patients had a lower HbA1C on the follow up visit compared with the first visit. At the first visit, pharmacists’ triage identified 15% of patients who had experienced hypoglycemia in last 30 days, as well as 44% of patients with adherence problems. Furthermore, because of the SMART methods in patient care and the suggested structure of counseling, patients who did not receive flu vaccination were identified. In the Program, 25% of the patients received flu vaccination on their first visit.

### 3.5. National Results Presented at Local and International Events

As a result of the Program, many new initiatives occurred on the national level in different countries. In Qatar, the hosts arrange collaborative agreements with endocrinologists, which clearly described the pharmacists’ service and the expected positive impact on the patients’, pharmacists’ and physicians’ levels in diabetes care. In Turkey, a project in cooperation with the Ministry of Health and the World Health Organization (WHO) on hypertension patients in three provinces was initiated, in order to effectively position pharmacists as a defined member of primary healthcare service teams.

In Montenegro, the collaboration between pharmacist and pulmonologists was initiated by the Chamber of Pharmacists, which is the national pharmacy regulator, and results will be presented at national conferences by the end of 2020. SMART Program results, patients’ stories, and testimonies of SMART Pharmacists were presented at national symposia and events in Oman, Turkey, Montenegro, Estonia, and separate SMART meetings to present the results are to be held in Serbia and Qatar. The Minister of Health in Oman recognized the Program as a successful and innovative program to be implemented in all sectors (public and private) of pharmacy in Oman.

Public health campaigns were organized in Turkey, Oman and Montenegro, based on the SMART activities, as well as television interviews and press conferences in Oman, Poland, Qatar, Montenegro, Kuwait, and Turkey. Social media activities were initiated in many countries, as well as advocacy activities in Ministries of Health, national medicines agencies, as well as WHO Regional Offices. The Program was presented at various national and international conferences, a few congresses of the International Pharmaceutical Federation (FIP)-most visibly at the FIP Regional Conference in Turkey in 2019, where it was recognized both by WHO and FIP as a successful model to implement advances and change in the pharmacy profession. Advocacy for the profession, based on locally collected evidence is one of the most important outcomes and impact of the Program in many countries.

Changes in the academic environment are also visible in some countries. In Northern Cyprus, based on SMART activities, a new course for pharmacy students was introduced in 2018 to develop life-long learning skills, reflection principles, and self-assessment for pharmacy students. The importance of preparedness for life-long learning in the context of pharmacy practice and education was emphasized. Self-awareness, self-directed learning skills and attitudes, beside other CPD associated skills, were adopted. A self-directed learning readiness tool was pilot tested and then provided for students in seven countries; publication of the results is pending.

At national meetings, pharmacists have reported that patients have shown interest in the new services provided under the Program and expressed greater trust in pharmacists’ advice. All host organizations agree or strongly agree that the Program leads to application of new knowledge, skills, and attitudes in practice, and that the Program has increased the motivation of pharmacists to advance pharmacy practice and education in their countries [53].

## 4. Discussion

### 4.1. Achieving the Results on Different Levels—Contributing Factors

The main purpose of the Program—to introduce changes in pharmacy education and practice—has been achieved. Results were visible on: (1) the individual level—increased levels of competencies of pharmacists (self-reported), maintenance of a learning portfolio, reflection and planning of learning, and especially the application of new skills and knowledge into practice and documentation of results; (2) the organizational level-increased Indicators of Quality of patient services in pharmacy, and introduction of new pharmacy services; (3) on the national level—improvements in the participation in CPD educational modules, public health campaigns, SMART Pharmacist meetings and symposia, patient safety improvements, and interprofessional collaboration; and (4) on the patient level-improved clinical outcomes, better quality of life, better inhalation technique, reduced salbutamol use, vaccination of chronic patients, adherence improvements, and improvement in Peak Flow Rates.

It appears that self-directed learning and the self-development approach were inspiring for pharmacists, both for those who were trainers and those who just participated in the Program. Self-assessment of their competencies and individual planning for their improvement were new skills for most of the participants included in the Program. Initially, it was planned that competency assessment would be done by peer assessment, but participating pharmacists expressed reservations about the proposed system of peer assessment. Consequently, the plan was dropped and after the pilot project in Turkey, it was decided that self-assessment would be used instead, despite the fact that results would be more subjective. Self-assessment is a skill itself, and it requires some experience, good understanding of the required standards and clinical procedures, objectivity, and self- motivation [21]. To be consistent, self-assessment was also used for QI assessment. As the Program matures, and the concept of assessment in practice becomes more acceptable, peer assessment may be considered again.

Some leaders in the Program stated that it reinforced the enthusiasm for the profession and that this new type of training with a strong focus on self-development for pharmacists was welcomed. A clear need for new models in pharmacy education, including this approach, was identified. Based on some hosts’ feedback, “*Learn Today—Apply Tomorrow*” was the most attractive and inspiring idea that came out of the Program, as it summarizes the importance of learning to impact not only knowledge, but the skills, values, and performance of pharmacists. It was anticipated that those changes would be visible as improvements in direct patient care with clinical and financial outcomes, as well as enhancing pharmacist–patient relationships. Furthermore, the Program allowed participants to identify the barriers and challenges to advancing pharmacists’ roles in patient care areas, especially connected with chronic, non-communicable diseases.

### 4.2. Facilitators and Barriers to Implementation

Some experiences in the implementation of the Program were important to help to understand how the Program could be more successful. One was the identification of the national context, and adjusting the Program to the country-specific needs, challenges, policies, and systems. Understanding local priorities and building national education teams were seen as an incentive, and embraced by the hosts. Another facilitator was the strong leadership, influence, and clear vision of the local host organization in how to mobilize the workforce, enhance the network of SMART Pharmacists, and develop supportive tools such as technology applications, social media announcements, presentations of best practices, and recognition of the champions of the Program. Some countries have introduced certificates and SMART Pharmacist badges to recognize and motivate the participants to achieve results. This was very important to facilitate the process.

Starting with a Master Class or conducting an Innovation Workshop can both be successful ways to initiate the Program, as long as interactive communication is included and enough key stakeholders are invited and participate. In some countries, like Jordan and Kuwait, the hosts reported that the Innovation Workshop was the first time for all the key stakeholders in the pharmacy profession to meet in one room and participate in an important discussion about pharmacy education and practice in their country.

Academic institutions, national professional institutions and organizations, and chambers have made the most progress with the implementation of the Program, and private companies the slowest, but there were also country-specific circumstances that influenced the development of the Program. Maintaining momentum at certain times was crucial to not losing impetus in the change management process, and in some countries, there were challenges in this regard. One observation from all countries was that the Program is introducing changes, so the change management strategy should be clear, and the situation mature enough for change to be successfully introduced. The implementation process is complex, involving a range of internal and external factors. As demonstrated before, facilitators should be used in a multi-level strategy to integrate professional services into the community pharmacy business, engaging pharmacists and their staff, policy makers, educators, and researchers [4,54].

The main barrier in the implementation of the Program was the resistance of participants to collect and record the data, both their own (self-assessments) and that of patients. Those challenges were expected, and different strategies were used to try to overcome them. A key purpose of the Program was to motivate pharmacists to measure and document the results of application of their learning and to demonstrate the impact of pharmacists’ interventions, both on the patient and pharmacist level. Only in this way, can pharmacists be recognized as SMART. Some host organizations did not clearly communicate this principle to the participants at the beginning; therefore, the data collection process was slow and/or incomplete in many cases.

In some countries, the Program experienced the challenge of trainers reducing the educational modules to be shorter and simpler, usually excluding or reducing the CPD Module. In those cases, the implementation of the Program was compromised. Understanding the principles of CPD as a new approach to self-directed learning, and keeping all stages of the CPD cycle active was crucial to motivate pharmacists and facilitate the implementation of the new educational model. Pre-reading was also introduced as a new approach to learning, but it was embraced by only a minority of pharmacists.

These findings align with previous systematic analysis of internal barriers, including: current organizational culture of the pharmacy; lack of an internal implementation champion; lack of priorities and goals; inappropriate layout in pharmacies (including the lack of a counseling room); lack of appropriate technology and resources; and lack of bibliographic resources and medicines- information support/assistance. At the pharmacy staff level: lack of leadership; lack of staff awareness on the relevance of the service; lack of priority to implement the service; inadequate workflow; and lack of staff training to provide the service were identified as the major barriers [55].

It is also critical to understand—and adjust strategies accordingly—that pharmacists will embrace or resist change differently. One of the most cited models in explaining innovation adoption is Rogers’ innovation curve [1,2]. The authors used Rogers’ model to describe how change can be accepted or resisted in the Pharmacy profession [4]. Importantly, it must be recognized that after the *Innovators* and *Early Adopters* and before the *Early Majority*, there is a “chasm” that must be crossed. This is invariably where otherwise good ideas fail, because the initial momentum and enthusiasm for change cannot be sustained when implementing change becomes more difficult. For this reason, the next stages of implementation of the SMART Program could be the most challenging, as in most countries the Program has only reached the first two categories of pharmacists so far, namely those who readily embrace change, even if it is challenging.

### 4.3. Impact of Communication Strategy to the Implementation

Social media, networking, motivational follow-up meetings, letters, and open communication facilitated the change process in many countries. Without continuing communication between originators of the Program and leaders of the host organization, as well as disseminated communication to the coordinators, trainers and participants, sustained change would not be possible. The motivation and courage of the local hosts sometimes decreased due to the slow or low response rates of participants, and sometimes even political circumstances. Experience shows that introducing this change was not an easy or straightforward process, but also that it was manageable, possible, and successful. Most importantly, the Program was and still is open to all pharmacists and professional stakeholders who desire to be a part of it. The originators of the Program introduced this inclusive and motivating approach to all host organizations and, once accepted, it was and remains one of the most important advantages of the implementation process of the Program.

## 5. Conclusions

Seeing the impact of their learning—as the application of expanded professional services changed and improved clinical outcomes, patient safety, and quality of life—was a huge motivating factor and inspired participating pharmacists to want to learn and change more. In this way, the ultimate purpose of CPD—repeated cycles of learning, enhancement of competence, and application of learning (performance)—is realized for the benefit of the practitioners, their organizations, and patients. According to Madden and Mitchell, these latter outcomes define the essence of CPD [56]. In addition to the countries where the Program has been introduced, many others have indicated a need and interest to implement the Program, and it is anticipated that the expansion of the Program will be possible and beneficial for many.

It was recognized early in the Program’s development in Turkey that the key issue would be change management. The concepts themselves (CPD, etc.) were not new and not complicated. What would be needed was strategic profession-wide leadership to take the globally adopted pharmaceutical care vision and make it a national reality. In some countries, the Program brought together key opinion leaders in the profession for the first time. So far, the Program has probably only been able to reach the Innovators and Early Adopters within the profession. Between these groups and the Early Majority, there is a “chasm.” It is at this stage that many otherwise good initiatives fail. The true test of the SMART Program and its sustainability, therefore, will be whether or not the chasm can be bridged to bring about deep pervasive change. It will certainly take commitment and resilience by those leading the national initiatives (Kotter’s seventh step of successful change) [49].

To make a parallel statement about education to the one made by Doug Hepler in 2010 about practice [6], “every educational activity must pass the test of whether it will facilitate and motivate pharmacists to improve the safety and effectiveness of drug therapy and, ultimately, our patients’ quality of life” [57]. The SMART Pharmacist Program is designed and intended to be a sustainable, evidence-based educational initiative with all elements of the CPD cycle, including application and impact on everyday pharmacy practice. When successfully implemented, with strong national leadership and participation from all key stakeholders, the Program has been shown to help to motivate and empower pharmacists to: enhance their competencies, expand their professional and patient-centered services, improve the quality of the services they provide, assume greater responsibility for the outcomes of patient care, and ultimately improve patient’s quality of life. It is time for Doug Hepler’s “dream” of Pharmaceutical Care to become a reality.

## Figures and Tables

**Table 1:** Levels of evaluation for outcomes of continuing education activities.

Level of Evaluation	Evaluated Outcome	Status
0	None	In some countries, no expectation or requirement, therefore no meaningful participation
1	Participation	Measured in most countries and professions; hours of participation transferred to credits, points, or units
2	Satisfaction	Measured by most continuing education (CE) providers
3	Learning	Measured by some CE providers
4	Performance	Measured in the SMART Pharmacist Program (both performance of the pharmacist and results on the patient level)
5	Patient health	
6	Population health	There are plans to measure this in future stages of the Program

**Table 2:** Countries and activities included in the SMART Program.

	Country	Program Started	Partner/Host Organization(s)	Master Class to Introduce Program	Innovation Workshop	TTT CPD Module	TTT Clinical Module	Training for Pharmacists by SMART Trainers	SMART Leadership Program	SMART Student/Preceptor/Educators/Program	Follow-Up SMART Presentation of Results at Other Events
1	Turkey	2014	Turkish Pharmacists’ Association	x	x	x	x	x	x	x	x
2	Northern Cyprus	2015	Near East University	x					x	x	
3	Spain	2015	Faculty of Pharmacy, University of Valencia	x						x	
4	Montenegro	2016	Pharmaceutical Chamber	x		x	x	x			x
5	India	2016	Indian Association of Colleges of Pharmacy	x					x	x	
6	Kuwait	2016	Life Sciences Academy; Kuwait CPD Committee		x	x	x	IP			
7	Romania	2017	Hospital Pharmacists’ Association	x							
8	Oman	2017	Directorate General of Medical Supplies, MoH	x		x	x	x			x
9	Egypt	2017	Children’s Cancer Hospital 57357	x	x	IP	IP	IP	IP	x	
10	Estonia	2017	Institute of Pharmacy, University of Tartu	x		x	x	IP			IP
11	Jordan	2017	Jordanian Pharmacists Association		x	x	x	x			
12	Indonesia	2018	Indonesian Pharmacists Association	x	x	x	x				
13	Poland	2018	Pharmaceutical Chamber	x	IP	IP	IP	IP	IP		
14	Armenia	2018	PharmProgress		x						IP
15	Qatar	2018	Hamad Medical Corporation		x	x	x	x	IP		IP
16	Serbia	2019	Pharmaceutical Chamber		x	x	x	IP			IP
17	Mauritius	2020	JSS University	x	IP					IP	
**Total X**				**12**	**8**	**9**	**9**	**5**	**3**	**5**	**3**
**Total IP**				**0**	**2**	**2**	**2**	**5**	**3**	**0**	**4**

Legend: X = Completed; IP= In Progress; TTT = train-the-trainer.

**Table 3:** Numbers of trained pharmacists in the Program.

	Country	SMART Trainers	Pharmacists Trained in CPD Module	Pharmacists Trained in Asthma and COPD Module	Pharmacists Trained in Pharmacists Patient Care Process Module	Pharmacists Trained in Diabetes Module	Pharmacists Trained in Hypertension and Dyslipidemia Module
1	Turkey *	341	5474	2761	820 *	3997 *	1265 *
2	Montenegro	12	51	51			
3	Kuwait	5	5	5			
4	Oman	89	89	61	35	28	28
5	Estonia	45	100		100		
6	Jordan	70	80				
7	Indonesia	80	80		80		
8	Qatar	39	144	21		144	
9	Serbia	40	40	37			
Total # Pharmacists Trained		**721**	**6063**	**2936**	1035 *	4169 *	1293 *

* Data from Turkey is from the SMART Program and Rehber Eczanem (derived from the SMART Program).

**Table 4:** Pharmacist and patient data collection and interpretation.

	Country	Pharmacist Data Collected	Patient Data Collected	Interpreted Results show Improvement	Data Published as National Case Report	Data Presented to Ministry of Health
1	Turkey	x	x	x	IP	x
2	Montenegro	x	x	x	IP	x
3	Oman	x	x	x	IP	x
4	Egypt	x				
5	Estonia	x	IP	IP		
6	Jordan	x	IP	IP		
7	Indonesia	x				
8	Poland	IP				
9	Qatar	x	x	x	IP	IP
10	Serbia	x	IP	IP	IP	IP
	**Total X**	**9**	**4**	**4**	**0**	**3**
	**Total IP**	**1**	**3**	**3**	**5**	**2**

Legend: X = Completed; IP= In Progress.
